# Trends in cardiovascular disease incidence among 22 million people in the UK over 20 years: population based study

**DOI:** 10.1136/bmj.q2381

**Published:** 2024-10-28

In this paper by Conrad and colleagues (*BMJ* 2024;385:e078523, doi:10.1136/bmj-2023-078523, published 26 June 2024), Werner Budts was based at the Department of Cardiovascular Sciences, KU Leuven, Leuven, Belgium and at Congenital and Structural Cardiology, University Hospitals Leuven, Belgium (affiliations 2 and 7 [not 1 and 7], respectively). The paper has been updated.

